# Supplementary material for: SIMPLseq: a high-sensitivity Plasmodium falciparum genotyping and PCR contamination tracking tool
Source: Malar J. 2026 Feb 3;25:131. doi: 10.1186/s12936-026-05796-1 (PMC12958562; doi:10.1186/s12936-026-05796-1)
Supplement: Supplementary file 1 — Supplementary Material 1. [file 12936_2026_5796_MOESM1_ESM.pdf]

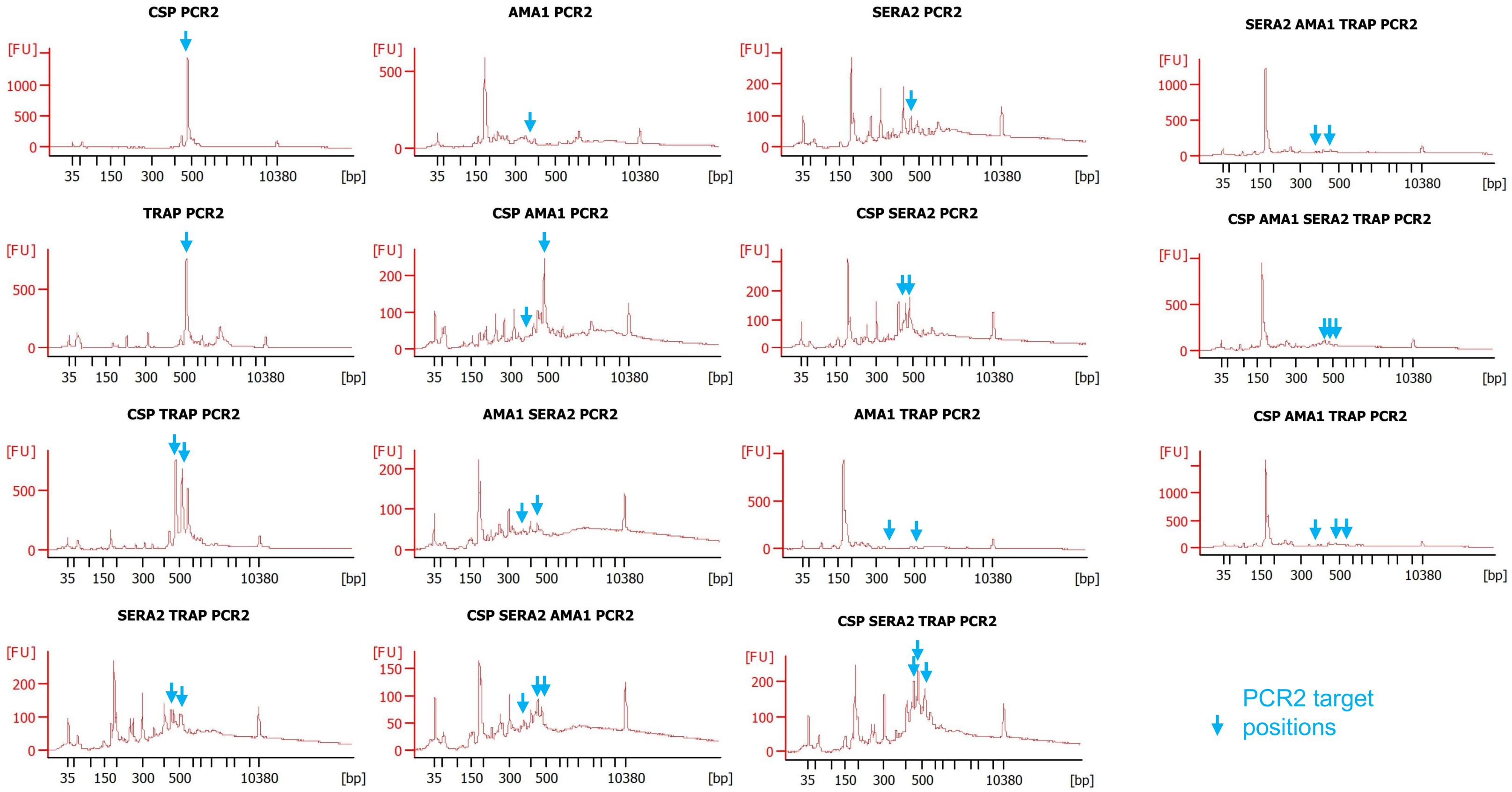

Supplementary Figure 1. PCR2 product traces for 4CAST and 4CAST subsets prior to bead-based clean-up.

Each trace represents a pool of 5 replicates using 1000 parasites/µl in whole human blood. Note large 190 bp peak and other abundant off-target amplification for reactions involving SERA2 or AMA1 targets. CSP and/or TRAP show higher reaction specificity.

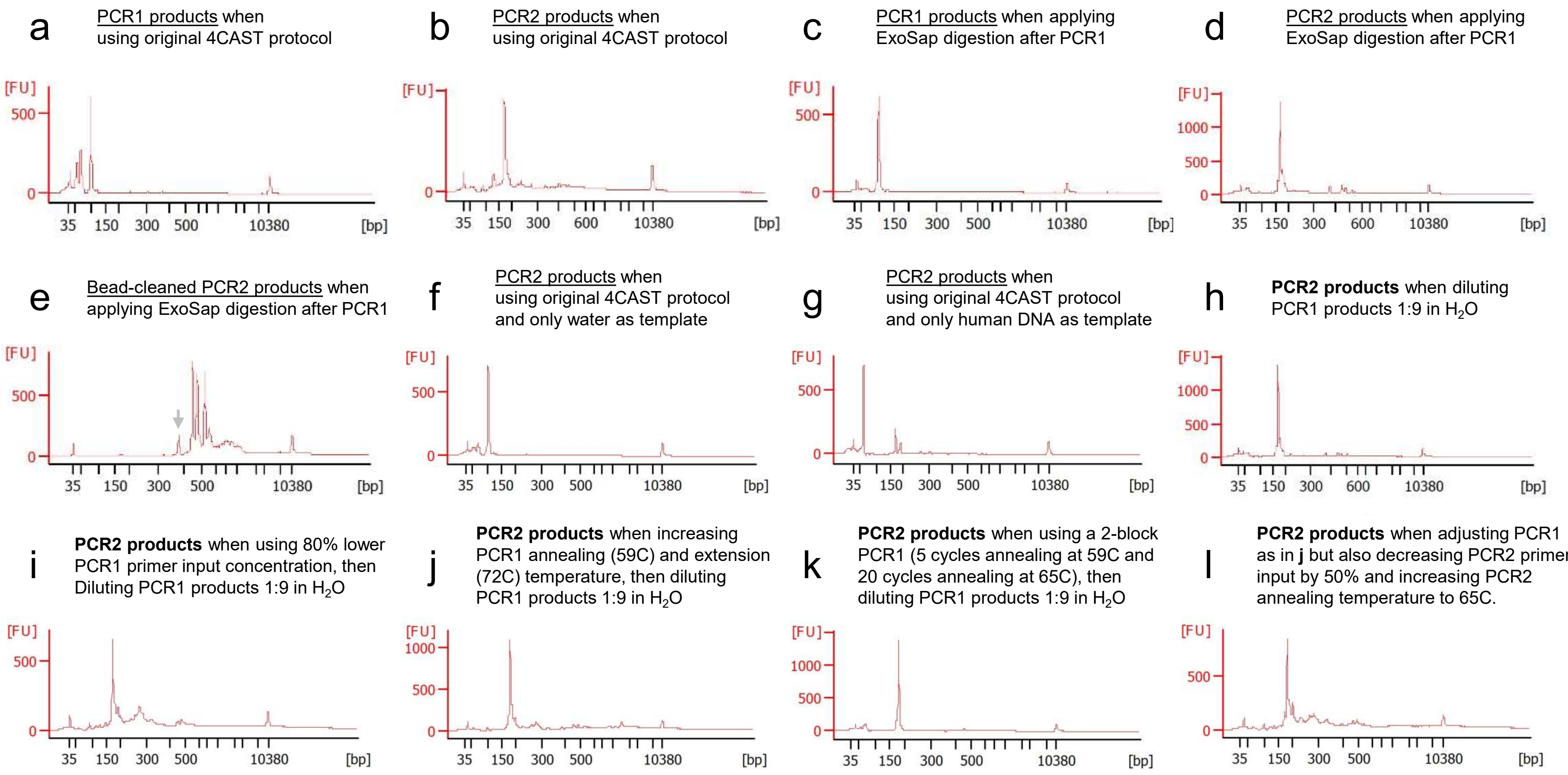

Supplementary Figure 2. PCR1 and PCR2 product traces (Bioanalyzer) for original and modified 4CAST protocols.

All positive traces represent the use of 16 parasites/µl in whole human blood (5 replicates, pooled) as initial PCR1 input. **a-b**) PCR1 and PCR2 products (prior to bead-based clean-up) via original 4CAST protocol (LaVerriere et al. 2022). **c-e**) PCR1 and PCR2 products (before and after bead-based clean-up) when ExoSap digestion is applied after PCR1. This digestion removes <75 bp fragments from PCR1 products and may slightly improve PCR2 target intensities, but off-target products still form abundantly near 190 bp. Off-target clean-up is difficult to achieve without eroding 4CAST targets, especially AMA1 (arrow). **f-g)** Off-target PCR2 products near 190 bp are not as abundant when only water or human DNA is used as PCR1 input. Parasite DNA may therefore be key to this artefact. **h-l)** PCR2 products (prior to bead-based clean-up) for a subset of other attempts (see labels) to improve the original 4CAST protocol. These modifications generally showed inferior clean-up results to those of the protocol involving ExoSap (**e**).

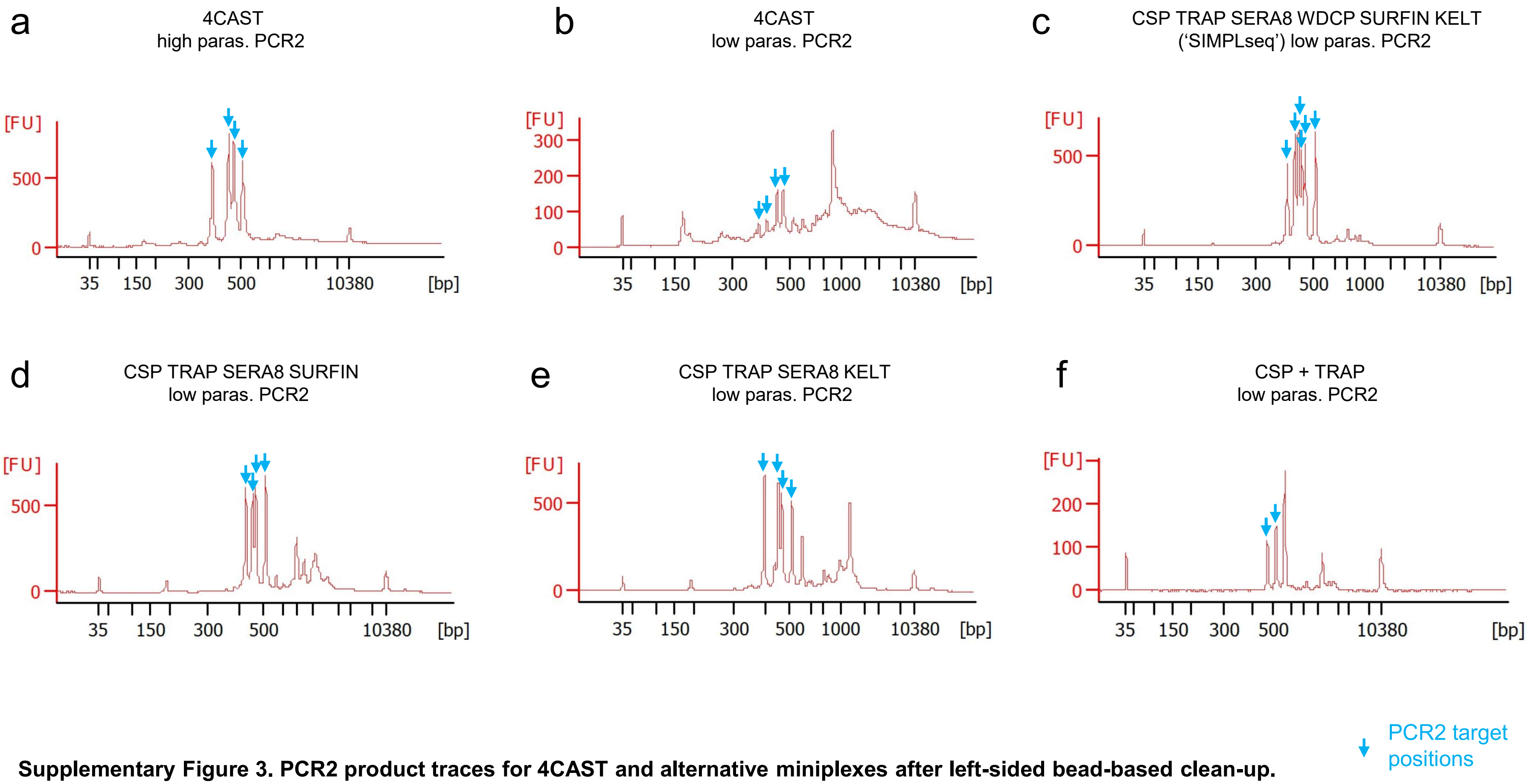

Supplementary Figure 3. PCR2 product traces for 4CAST and alternative miniplexes after left-sided bead-based clean-up.

a) 4CAST using 5000 parasites/µl in whole human blood (5 replicates, pooled).
b-f) 4CAST and alternative miniplexes using 0.125 – 8 parasites/µl in whole human blood (5 replicates per parasitemia level, pooled).

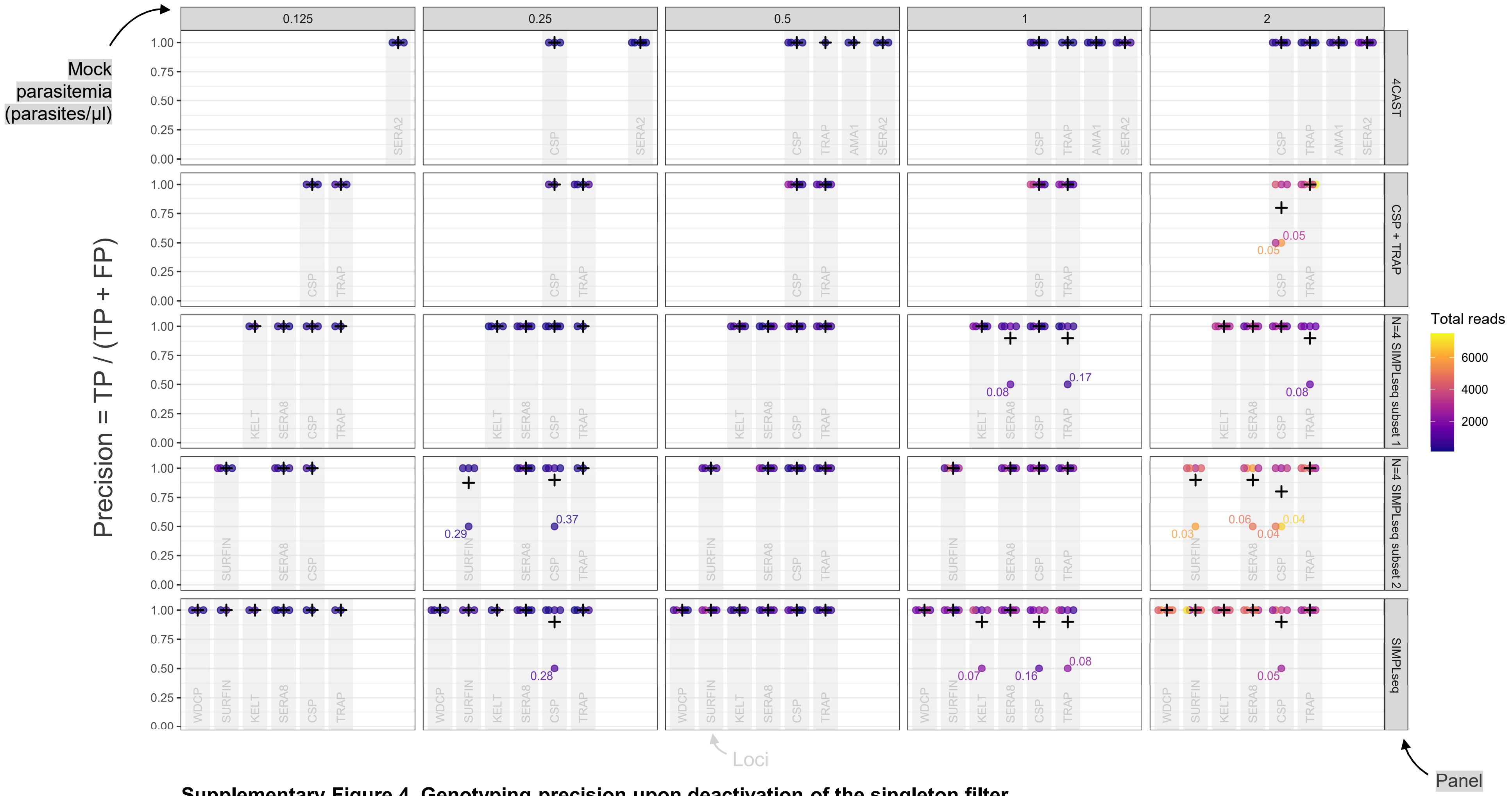

Supplementary Figure 4. Genotyping precision upon deactivation of the singleton filter.

Each point represents one of 5 replicates. Y-axis values represent precision (the proportion of detected haplotypes which contained the correct (Dd2-matching) sequence). For example, if a replicate occurs at y-axis value = 0.50, this means there was one additional, incorrect minor haplotype found in that replicate, i.e., 1 of 2 haplotype calls was incorrect; precision was therefore 50% for the replicate.

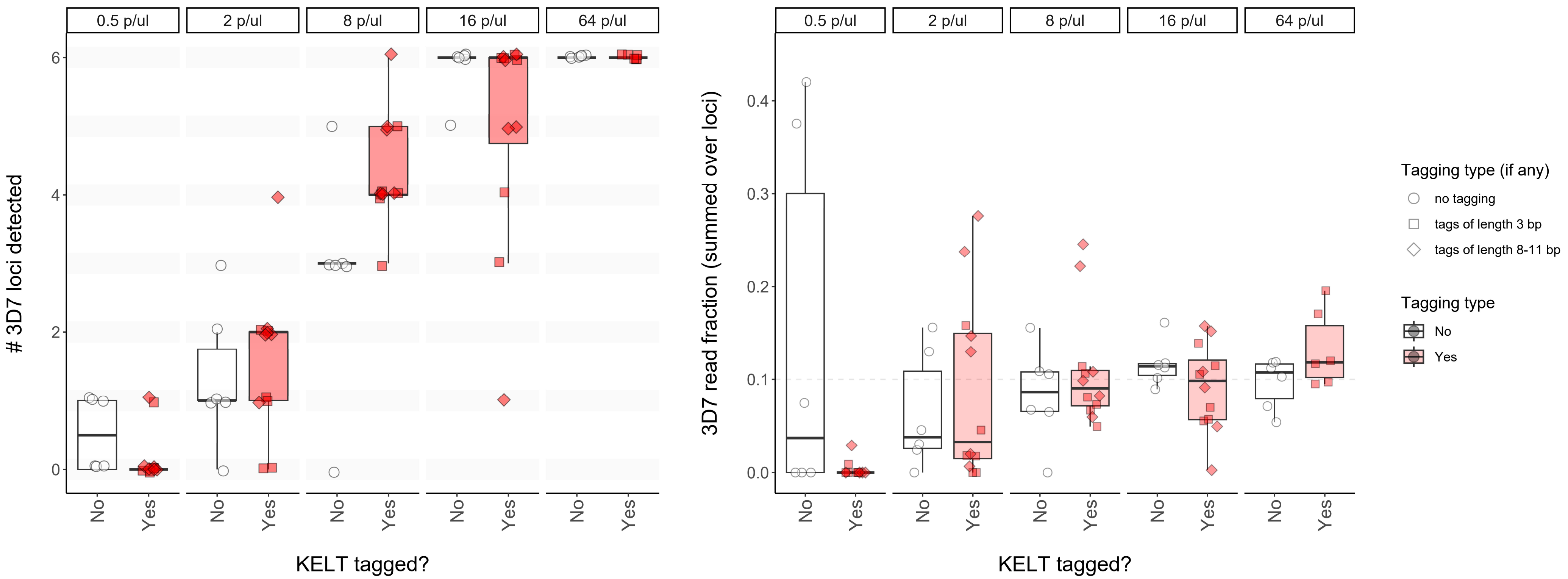

Supplementary Figure 5. Minor strain detection (untagged and KELT-tagged SIMPLseq applied to mock mixtures of 3D7 and Dd2 parasites).

Boxplots represent median and interquartile ranges for **a)** the number of 3D7-matching loci and **b)** the 3D7-matching read fraction (i.e., the number of read-pairs matching 3D7 divided by the number of read-pairs matching 3D7 or Dd2, across all loci) when applying SIMPLseq to mock mixtures containing 3D7 (10%) and Dd2 parasites (90%). The x-axis indicates whether the SIMPLseq reaction was untagged or included inline-barcoded primer pairs for the KELT locus. For KELT-tagged reactions (see also red fill color), barcodes were either short (3 bp, square symbols) or longer in length (8-11 bp, diamonds). Reactions used parasitemias between 0.5 and 64 p/µl (see facetting left to right). A minimum locus depth filter of 10 was used in the analysis.

	1	2	3	4	5	6	7	8	9	10	11	12
A												
B	*GTA/CAT*	*GTA/CAT*	*GTA/CAT*	*GTA/CAT*	*GTA/CAT*	*GTA/CAT*	*GTA/CAT*	*GTA/CAT*	*GTA/CAT*	*GTA/CAT*	*GTA/CAT*	*GTA/CAT*
C	ATG/CAT	ATG/CAT	ATG/CAT	ATG/TCA	ATG/TCA	ATG/TCA	ATG/CAT	ATG/CAT	ATG/CAT	ATG/TCA	ATG/TCA	ATG/TCA
D	*GTA/CAT*	*GTA/CAT*	*GTA/CAT*	*GTA/CAT*	*GTA/CAT*	*GTA/CAT*	*GTA/CAT*	*GTA/CAT*	*GTA/CAT*	*GTA/CAT*	*GTA/CAT*	*GTA/CAT*
E	ATG/CAT	ATG/CAT	ATG/CAT	ATG/TCA	ATG/TCA	ATG/TCA	ATG/CAT	ATG/CAT	ATG/CAT	ATG/TCA	ATG/TCA	ATG/TCA
F	*GTA/CAT*	*GTA/CAT*	*GTA/CAT*	*GTA/CAT*	*GTA/CAT*	*GTA/CAT*	*GTA/CAT*	*GTA/CAT*	*GTA/CAT*	*GTA/CAT*	*GTA/CAT*	*GTA/CAT*
G	ATG/CAT	ATG/CAT	ATG/CAT	ATG/TCA	ATG/TCA	ATG/TCA	ATG/CAT	ATG/CAT	ATG/CAT	ATG/TCA	ATG/TCA	ATG/TCA
H	*ATG/CAT*	*ATG/CAT*	*ATG/CAT*	*ATG/TCA*	*ATG/TCA*	*ATG/TCA*						

PCR1 input:

- 64 p/ul Dd2
- 64 p/ul 3D7
- H2O

- no reaction

Supplementary Figure 6. Plate layout for the deliberate contamination experiment using an inline-barcoded sentinel locus.

This plate layout was used to enact 3 different deliberate contamination events:

Event 1: 0.3 µl pipetted from row B into row C just before sealing for PCR1-thermocycle;

Event 2: 0.3 µl pipetted from row D into row E just after PCR1-thermocycle;

Event 3: 0.3 µl pipetted from row F: first, all digestions products correctly pipetted into the PCR2 buffer plate as normal, then an additional transfer of digestion product from row F).

Recipient wells represent either 3D7 (gold fill) or water (white fill) as initial PCR1 input template. All donor wells represent Dd2 (blue fill) as initial PCR1 input template. Asterisks indicate wells which were not intended to receive contamination. The inline barcoding used for recipient wells compares to that of donor wells in one of two ways: Recipient wells in columns 1, 2, 3, 7, 8, and 9 are designated with partially unique barcode pairs with respect to their donor wells, e.g., ATG/CAT vs. GTA/CAT. Recipient wells in columns 4, 5, 6, 10, 11, and 12 are designated with dually unique barcode pairs with respect to their donor wells, e.g., ATG/TCA vs. GTA/CAT.

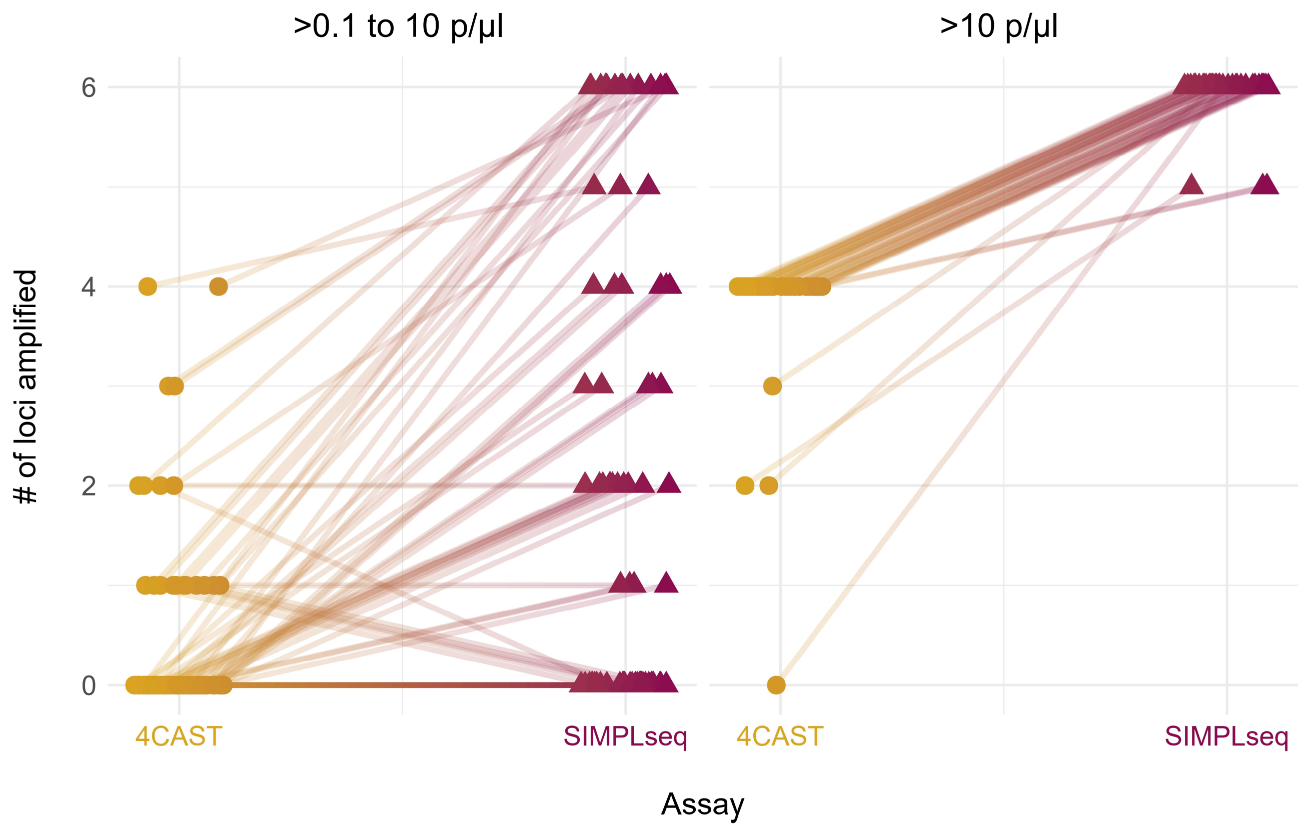

Supplementary Figure 7. 4CAST versus SIMPLseq (KELT-tagged) application to pediatric cohort samples from Mali.

4CAST data were obtained for 118 qRT-PCR-positive samples from Kayentao et al. 2024 (NEJM). Each sample was re-assayed using SIMPLseq (including inline-barcoded primer pairs for the KELT locus). Lines connect each 4CAST vs. SIMPLseq result (one line per dually-assayed sample). The y-axis indicates the number of detected loci.
No minimum locus read-depth threshold is used in this analysis.
